# Supplementary material for: Structural basis for phosphatidylcholine synthesis by bacterial phospholipid N-methyltransferases
Source: J Biol Chem. 2025 Apr 11;301(5):108507. doi: 10.1016/j.jbc.2025.108507 (PMC12139422; doi:10.1016/j.jbc.2025.108507)
Supplement: Figure S1–S5 [file mmc1.pdf]

Structural basis for phosphatidylcholine synthesis by bacterial phospholipid *N*-methyltransferases

Yasunori Watanabe[1] *, Hiroyuki Kumeta[2], Seiya Watanabe[3,4,5]

From [1]Faculty of Science, Yamagata University, 1-4-12 Kojirakawa-machi, Yamagata 990-8560, Japan; [2]Graduate School of Life Science, Hokkaido University, Kita 10, Nishi 8, Kita-ku, Sapporo, Hokkaido 060-0808, Japan; [3]Faculty of Agriculture, Ehime University, 3-5-7 Tarumi, Matsuyama, Ehime, 790-8566, Japan; [4]Department of Bioscience, Graduate School of Agriculture, Ehime University, 3-5-7 Tarumi, Matsuyama, Ehime, 790-8566, Japan; [5]Center for Marine Environmental Studies (CMES), Ehime University, 2-5 Bunkyo-cho, Matsuyama, Ehime 790-8577, Japan.

*Corresponding author: Yasunori Watanabe; Faculty of Science, Yamagata University, 1-4-12 Kojirakawa-machi, Yamagata 990-8560, Japan;
yasunori@sci.kj.yamagata-u.ac.jp; Tel. +81 23 628 4529.

Materials included:

Figure S1

Figure S2

Figure S3

Figure S4

Figure S5

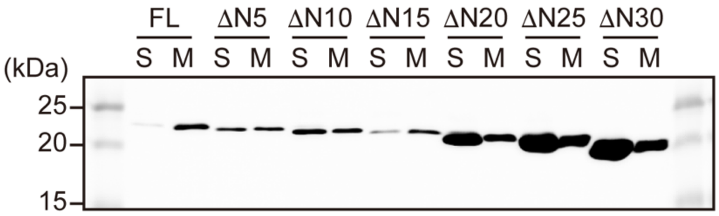

Figure S1. Membrane-binding activity of the N-terminal region lacking AtPmtA mutants
Soluble (S) and membrane (M) fractions were separated from *E. coli* cells expressing full-length AtPmtA (FL) or the N-terminal truncated mutants of AtPmtA (ΔN5, ΔN10, ΔN15, ΔN20, ΔN25, and ΔN30) and subjected to SDS-PAGE, followed by immunoblotting with anti-6x histidine antibody.

A

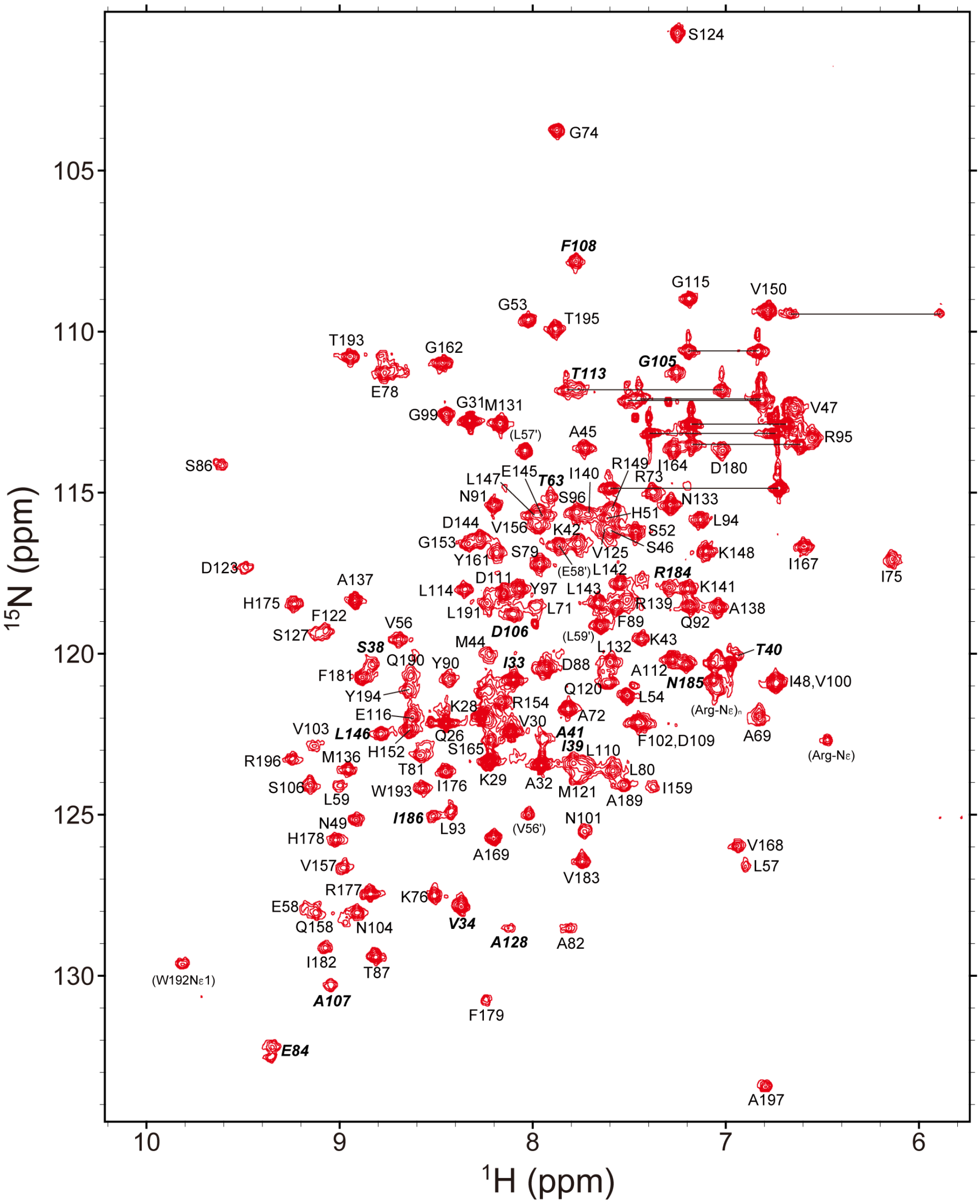

26 QPKKVGAIVPTSSITAKKMASVINPHSGLPVLELGPGTGVITKAILARGIKPESL 80
81 TAIEYSTDFYNQLLRSYPGVNFVNGDAFDLDATLGEHKGQMFDSVISAVPMLNFP 135
136 MAARIKLLDELLKRVPHGRPVVQISYGPISPIVAQPHLYHIRHFDFIVRNIPPAQ 190
191 LWTYTRA 197

B

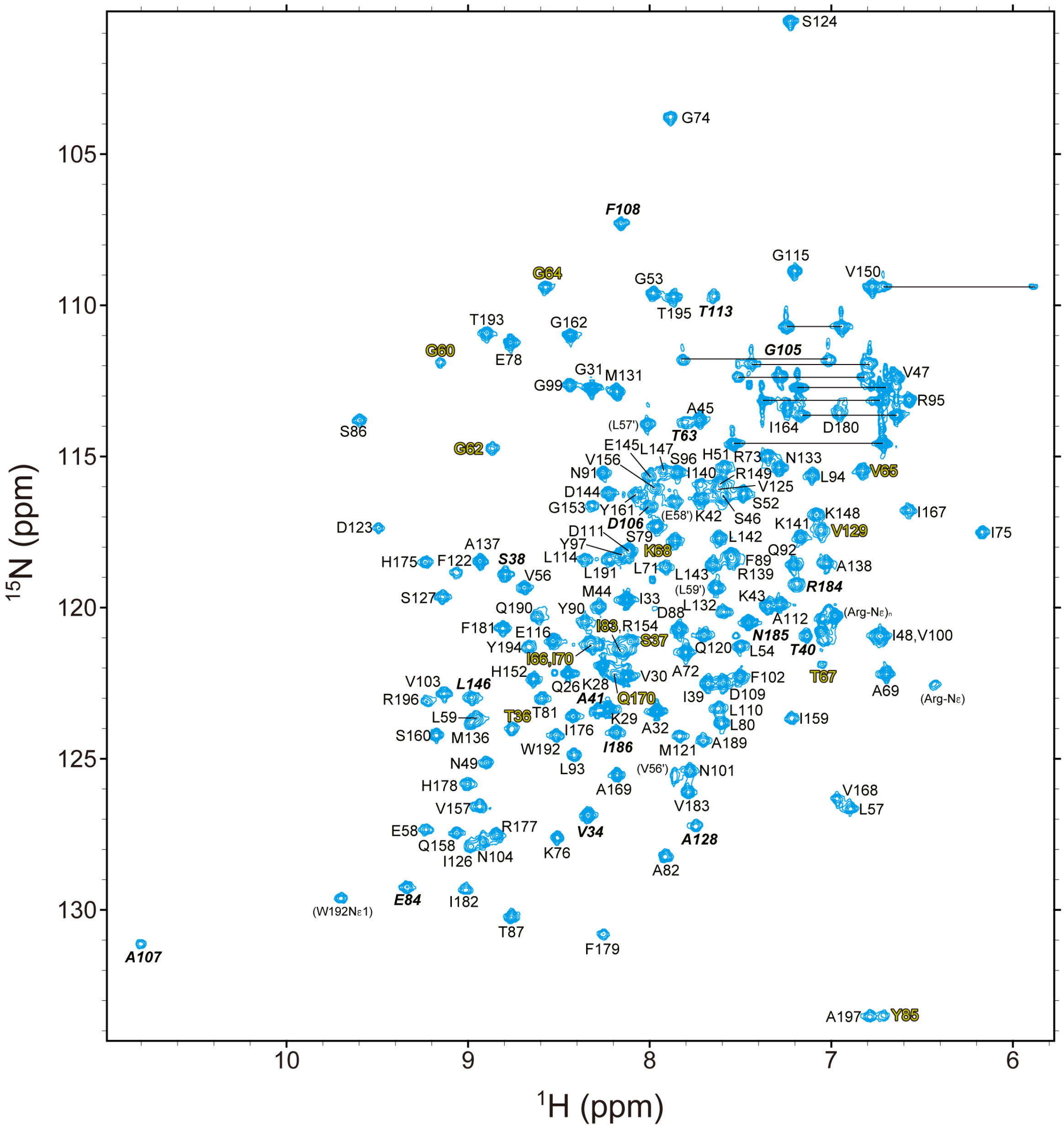

26 QPKKVGAIVPTSSITAKKMASVINPHSGLPVLELGPGTGVITKAILARGIKPESL 80
81 TAIEYSTDFYNQLLRSYPGVNFVNGDAFDLDATLGEHKGQMFDSVISAVPMLNFP 135
136 MAARIKLLDELLKRVPHGRPVVQISYGPISPIVAQPHLYHIRHFDFIVRNIPPAQ 190
191 LWTYTRA 197

C

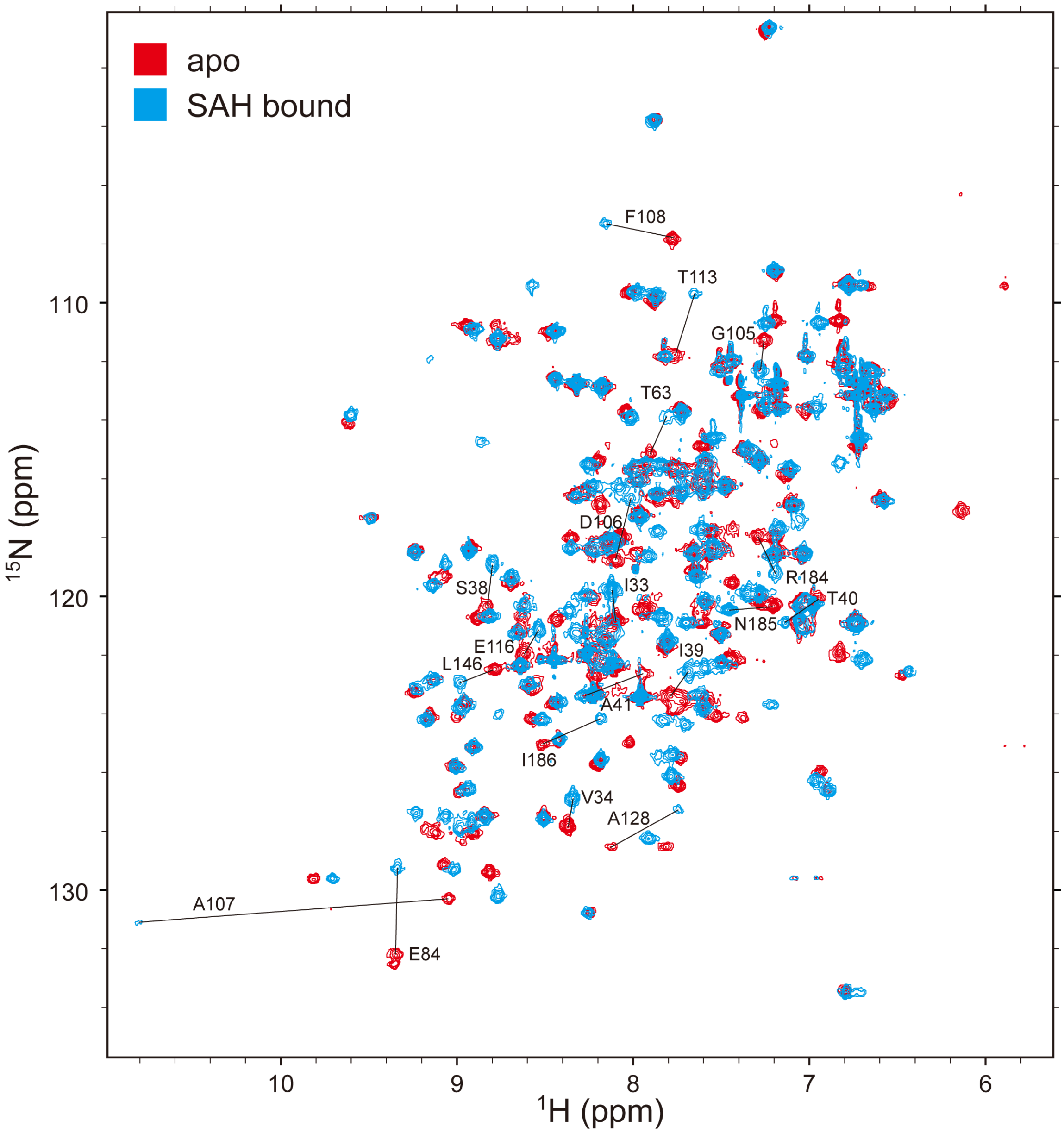

Figure S2. Assigned ^{1}H-^{15}N HSQC spectra of apo and SAH bound AtPmtAΔN25
(A-B) [^{1}H-^{15}N] HSQC spectra with resonance assignments of 300 μM AtPmtAΔN25 in the absence (A) and presence of 3 mM SAH (B). The residues with significant chemical shift changes upon binding to SAH are labeled in italic. The residues whose signals appeared upon binding to SAH are labeled in yellow. Minor form assignments are indicated by the prime symbol ('). Amino group resonances of Asn and Gln side chains are connected by horizontal lines. Amino acid sequence of AtPmtAΔN25, with assigned residues in black and unassigned residues in gray, is shown below each spectrum. (C) Overlay of the [^{1}H-^{15}N] HSQC spectra with resonance assignments of 300 μM AtPmtAΔN25 in the absence (red) and presence of 3 mM SAH (blue). The residues with significant chemical shift changes upon binding to SAH are indicated.

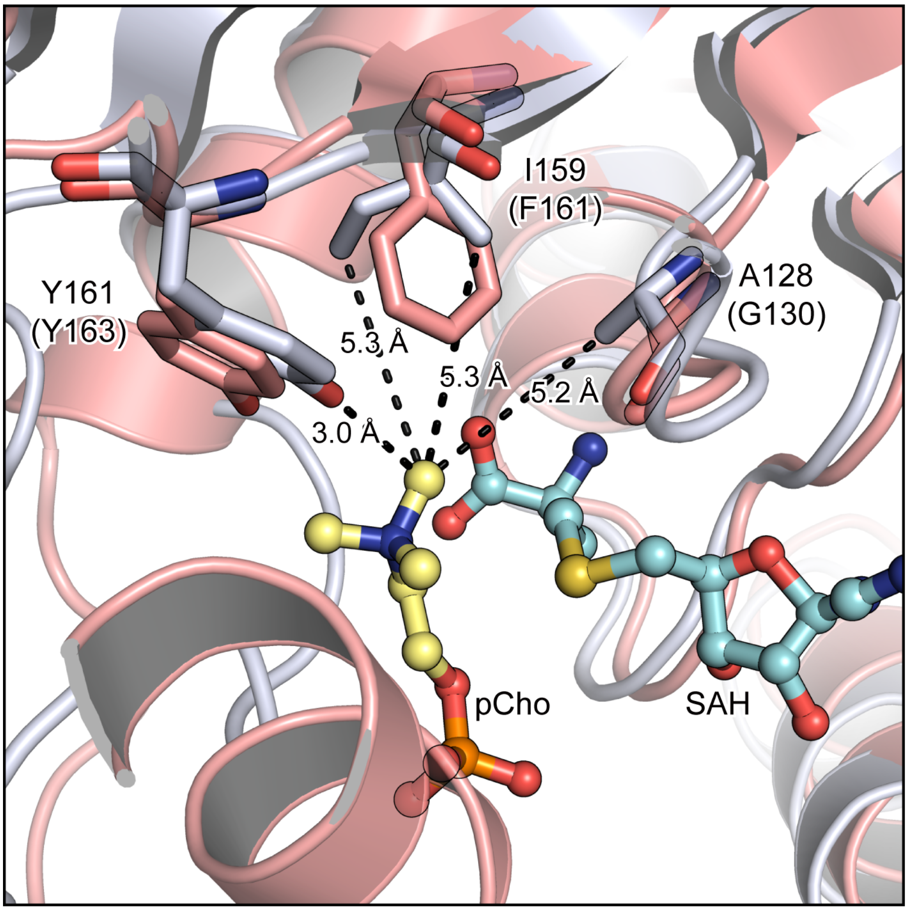

light blue: AtPmtAΔN25-SAH (This study)
pink: BdPmtA (AlphaFold 2)

Figure S3. Substrate binding sites of AtPmtA and BdPmtA

Superposition of the docking model of the AtPmtAΔN25-SAH complex with pCho and the AlphaFold 2-predicted structure of BdPmtA. AtPmtAΔN25 and BdPmtA are colored in light blue and pink, respectively. Ala128, Ile159, and Tyr161 in AtPmtA and Gly130, Phe161, and Tyr163 in BdPmtA are shown in stick form. BdPmtA residues are indicated in parentheses. The distances between the methyl group of pCho and side chains of Ala128, Ile159, and Tyr161 in AtPmtA.

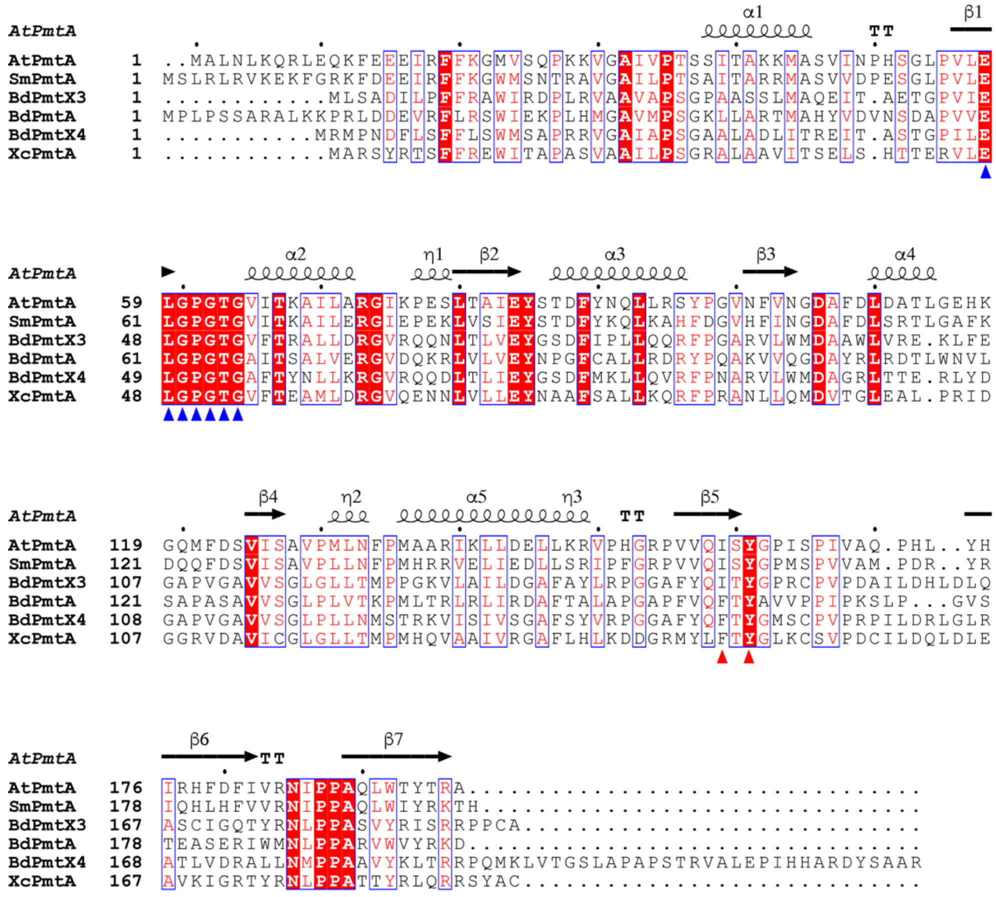

Figure S4. Sequence alignment of S-type Pmt homologs

Multiple-sequence alignment of AtPmtA with S-type Pmt homologs from *Sinorhizobium meliloti* (SmPmtA), *Bradyrhizobium diazoefficiens* (BdPmtA, BdPmtX3, and BdPmtX4), and *Xanthomonas campestris* (XcPmtA). Identical residues are shaded red, similar residues are colored red, and similar residues across groups are framed in blue. The secondary structure of AtPmtA is shown above the sequences. The conserved SAM-binding motifs are indicated with blue arrowheads. Ile159 and Tyr161 in AtPmtA are indicated with red arrowheads.

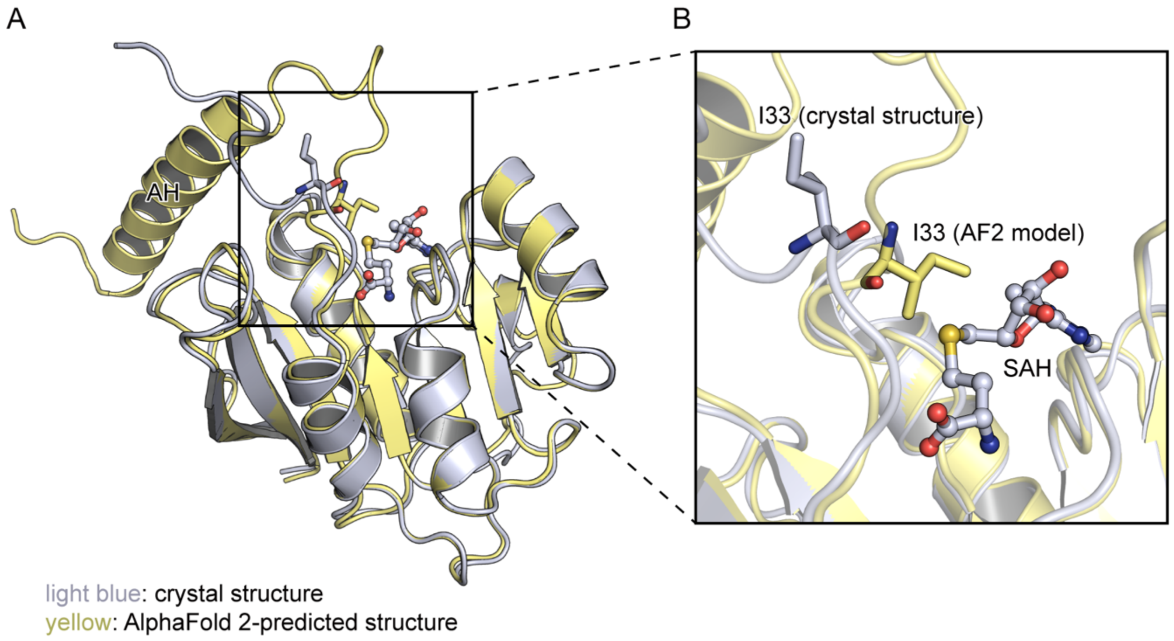

Figure S5. Comparison of AlphaFold 2-predicted and crystal structures of AtPmtA
(A) Superposition of the AlphaFold 2-predicted structure of AtPmtA (yellow) and the crystal structure of AtPmtAΔN25 (light blue). The N-terminal amphipathic helix of the AlphaFold 2-predicted structure of AtPmtA is labeled as AH. (B) The magnified view around Ile33 of AtPmtA. Ile33 is shown in stick form, whereas SAH is shown in stick and ball form.
